# Hydralazine target: From blood vessels to the epigenome

**DOI:** 10.1186/1479-5876-4-10

**Published:** 2006-02-28

**Authors:** Claudia Arce, Blanca Segura-Pacheco, Enrique Perez-Cardenas, Lucia Taja-Chayeb, Myrna Candelaria, Alfonso Dueñnas-Gonzalez

**Affiliations:** 1 Unidad de Investigación Biomédica en Cáncer, Instituto de Investigaciones Biomédicas (IIB)/Instituto Nacional de Cancerología, Universidad Nacional Autónoma de Mexico, Mexico City, Mexico; 2 Division of Clinical Research, Instituto Nacional de Cancerología, Mexico City, Mexico

## Abstract

Hydralazine was one of the first orally active antihypertensive drugs developed. Currently, it is used principally to treat pregnancy-associated hypertension. Hydralazine causes two types of side effects. The first type is an extension of the pharmacologic effect of the drug and includes headache, nausea, flushing, hypotension, palpitation, tachycardia, dizziness, and salt retention. The second type of side effects is caused by immunologic reactions, of which the drug-induced lupus-like syndrome is the most common, and provides clues to underscoring hydralazine's DNA demethylating property in connection with studies demonstrating the participation of DNA methylation disorders in immune diseases. Abnormalities in DNA methylation have long been associated with cancer. Despite the fact that malignant tumors show global DNA hypomethylation, regional hypermethylation as a means to silence tumor suppressor gene expression has attracted the greatest attention. Reversibility of methylation-induced gene silencing by pharmacologic means, which in turns leads to antitumor effects in experimental and clinical scenarios, has directed efforts toward developing clinically useful demethylating agents. Among these, the most widely used comprise the nucleosides 5-azacytidine and 2'deoxy-5-azacytidine; however, these agents, like current cytotoxic chemotherapy, causes myelosuppression among other side effects that could limit exploitation of their demethylating properties. Among non-nucleoside DNA demethylating drugs currently under development, the oral drug hydralazine possess the ability to reactivate tumor suppressor gene expression, which is silenced by promoter hypermethylation *in vitro *and *in vivo*. Decades of extensive hydralazine use for hypertensive disorders that demonstrated hydralazine's clinical safety and tolerability supported its testing in a phase I trial in patients with cancer, confirming its DNA demethylating activity. Hydralazine is currently being evaluated, along with histone deacetylase inhibitors either alone or as adjuncts to chemotherapy and radiation, for hematologic and solid tumors in phase II studies.

## Review

### Hydralazine as an antihypertensive

Hydralazine, a potent arterial vasodilator that reduces peripheral resistance directly by relaxing the smooth muscle cell layer in arterial vessels, has long been used for management of hypertensive disorders and heart failure [1,2]; nonetheless, its current use is limited nearly to hypertensive disorders during pregnancy [3,4]. Despite numerous studies with the drug, its mechanism of action has remained unknown but it is suggested that hydralazine may function by either modulating the effect of purine-like compounds released from sympathetic nerve endings, and/or by producing an altered Ca^2+ ^balance in vascular smooth muscle cells [5-7]. The majority of its effects are confined to the cardiovascular system. Decrease in blood pressure after hydralazine administration is associated with a selective decrease in vascular resistance in coronary, cerebral, and renal circulation, with a lesser effect in skin and muscle. Hydralazine lowers peripheral vascular resistance equally in supine and upright positions; it also lowers pulmonary vascular resistance and increases cardiac output causing mild pulmonary hypertension. [1,2].

Hydralazine is well absorbed through the gastrointestinal tract, but systemic bioavailability is low. Because the acetylated compound is inactive, the dose necessary to produce a systemic effect is higher in fast acetylators. N-acetylation of hydralazine occurs in bowel and/or liver. Hydralazine's half-life is 1 h and systemic clearance of the drug is approximately 50 mL/kg/min. Hydralazine rapidly combines with circulating α keto-acid to form hydrazones, and the major metabolite recovered from the plasma is hydralazine piruvic acid hydrazone. This metabolite possesses a longer half-life than hydralazine but does not appear to be very active. Systemic metabolism is dependent on hydroxylation followed by conjugation with glucoronic acid in liver, which is not dependent on acetylation rate; therefore, half-life does not differ to a great degree between slow and fast acetylators [8]. Hydralazine peak concentration in plasma and peak hypotensive effect of the drug occurs within 30–120 min of ingestion. Although its half-life in plasma is approximately 1 h, duration of the hypotensive effect can last as long as 12 h; there is no clear explanation for this discrepancy. The antihypertensive effect of hydralazine has no clear dose-response effects. The dose varies from 10 mg four times a day to 50 mg four times daily. After stabilization with multiple daily doses, a twice-daily dose regimen can be effective. Slow acetylators require a lower dose. For heart failure, recommended doses are higher (up to 800 mg daily or more); as a rule, 10–100 mg four times a day can be effective [9].

Two types of side effects occur after hydralazine use. The first type, an extension of hydralazine's pharmacologic effect, includes headache, nausea, flushing, hypotension, palpitation, tachycardia, dizziness, and angina pectoris; hydralazine can produce salt retention with congestive heart failure development. The second type of side effect is caused by immunologic reactions, of which the drug-induced lupus-like syndrome is the most common [8,9].

### Cancer epigenetics

Epigenetics can be defined as the study of mitotically and/or meiotically heritable changes in gene function that can not be explained by changes in DNA sequence. There are two epigenetic systems that affect animal development and fulfill the heritability criterion: DNA methylation, and the polycomb-trithorax group (Pc-G/trx) protein complexes; nevertheless, post-transductional histone modification possesses some attributes of an epigenetic process and as such are studied within this field [10]. Epigenetics is a well-established phenomenon that plays a major role in a diversity of biological processes such as embryonic development, cancer biology, and immune system response, among many others. The two most widely studied epigenetic changes are DNA methylation and histone acetylation; however, the picture is much more complicated than this, with new players coming into the scenario; these include, among others, the RNA interference phenomenon, proven to be implicated in transcriptional silencing through small duplex RNA molecules that recruit silencing complexes to the chromatin, and the BORIS (Brother of the Regulator of Imprinted Sites)/CTCF gene family, which is involved in epigenetic reprogramming events [11,12].

### DNA methylation

DNA methylation is a covalent chemical modification that mainly occurs at the cytosine ring, resulting in the addition of a methyl^CH3 ^group at the carbon 5 position. Because DNA is made up of four bases, 16 possible dinucleotide combinations can occur; hence, the CpG dinucleotide should have a frequency of 6%. Nonetheless, actual presence is only 5–10% of its predicted frequency. The human genome is not uniformly methylated and contains regions of unmethylated interspersed with methylated segment regions. In contrast to the remainder of the genome, smaller regions of DNA – denominated CpG islands, ranging from 0.5–5 kb, and occurring on average every 100 kb – possess distinctive properties. These regions are unmethylated, GC-rich (60–70%), have a ratio of CpG:GpC of at least 0.6, and thus show no suppression of dinucleotide CpG frequency. Approximately one half of all genes in humans have CpG islands, these present in both housekeeping genes and in genes with tissue-specific patterns of expression [13,14].

At least three functional DNA methyltransferases (DNMT) have been identified; the most abundant is DNMT1, or maintenance DNMT. This methyltransferase enzyme copies pre-existing methylation patterns onto the new DNA strand during DNA replication to ensure clonal transmission of lineage-specific DNA methylation patterns in a mammalian genome during replication. Thus, DNMT1 is targeted to replication foci, interacts with proliferating cell nuclear antigen, and favors methylating the hemimethylated form of CpG sites. It has been demonstrated that DNMT1 methylates hemimethylated DNA with high processivity and a fidelity of >95% on one strand of double-stranded DNA during a single processive run; nevertheless, DNMT1 methylates unmethylated DNA with much less processivity, suggesting that these inherent enzymatic properties of Dnmt1 play an essential role in the faithful and efficient maintenance of methylation patterns in the mammalian genome [15,16].

Other known functional methyltransferases include DNMT3a and DNMT3b, which are responsible for *de novo *methylation during embryogenesis; thus, these have been classified as *de novo *methyltransferases [17]. These enzymes are mainly responsible for introducing cytosine methylation at previously unmethylated CpG sites by at least three possible means: first, DNMT3 enzymes themselves might recognize DNA or chromatin via specific domains; second, DNMT3a and DNMT3b might be recruited through protein-protein interactions with transcriptional repressors or other factors, and third, the RNA-mediated interference (RNAi) system might target *de novo *methylation to specific DNA sequences [18].

In addition to DNMTs, the machinery of methylation includes demethylases, methylation centers that trigger DNA methylation, and methylation protection centers [19]. The effect of DNA methylation on gene transcription can be observed only within the context of chromatin remodeling players. DNA methylation can interfere directly with transcriptional factor binding, thus inhibiting replication [20], in addition to the ability of DNA methyltransferases DNMT1, DNMT3a, and DNMT3b to repress transcription in a methylation-independent manner [21]. Methyl-CpG-binding proteins, which can recognize methylated DNA, have been shown to associate with large protein complexes containing HDACs and chromatin-remodeling activities. It has also been suggested that DNA methylation could produce gene silencing by methyl-binding domain proteins that recruit histone methyltransferases, which methylate lysine 9 in histone H3 and subsequently repress gene transcription [22]. As a result, histones are deacetylated and gene transcription repressed.

### Histones and post-translational modifications

How double-strand DNA is packaged into the dynamic structure of chromatin is crucial for the transcriptional control process by regulating transcription factor accessibility to DNA regulatory sequences. Chromatin is constituted of nucleosomes, which are comprised of 146 base pairs of DNA wrapped around a core of two copies each of histones H2A, H2B, H3, and H4. The N-terminal tails of histones, which undergo post-translational modifications such as acetylation, methylation, ubiquitination, phosphorylation, and sumoylation, protrude from their originating nucleosome and may contact adjacent nucleosomes as well as chromatin-associated proteins. These interactions are responsible, at least in part, for regulating chromatin structure [23]. The most widely studied modification is acetylation, which plays diverse roles in nucleosome regulation. Lysine acetylation, for example, may decrease histone-DNA interaction and promote accessibility of DNA for transcription activation. Moreover, acetylation specifically can also regulate DNA replication, histone deposition, and DNA repair by mechanisms of recruiting proteins that have an acetyl-lysine binding module, the bromodomain, a 100-amino-acid conserved sequence found in many chromatin-associated proteins. Thus, bromodomains in histone acetyltransferases and nucleosome remodeling complexes orchestrate the succession of chromatin regulators at a target site [24,25]. Overall, lysine acetylation results in chromatin decompactation, greater access of DNA to transcription factors, and the presence of a transcriptionally active genomic locus. This post-translational modification depends on the net local balance between histone acetyl transferase (HAT) and histone deacetylase (HDAC) activities. In general, hyperacetylation is found in more decondensed euchromatin, whereas hypoacetylation is characteristic of more condensed heterochromatin [26,27].

### Epigenetic alterations and cancer

Abnormalities in DNA methylation have long been associated with cancer. Both hypo- and hypermethylation play prominent roles in carcinogenesis, and their contribution exhibits poorly defined boundaries. It has been known for some time that both alterations co-exist in cancer cells: malignant tumors exhibit global hypomethylation and regional hypermethylation. Whether one must precede the other or whether both should begin at the same time remains to be elucidated. In terms of carcinogenesis, initial observations were conducted on hypomethylation [28 29]; later, discovery of regional hypermethylation as a means to silence tumor suppressor gene expression gained the most attention [30].

### Silencing of tumor suppressor genes

Observations that tumor suppressor gene function can be disrupted not only through structural changes (mutation and deletion) but also by lack of promoter hypermethylation-associated expression positioned tumor suppressor gene epigenetic silencing as a well-established oncogenic process [31]. The first suppressor gene known to be hypermethylated and silenced was *Rb *[32]. This finding was soon followed by multiple publications describing similar findings for a variety of tumor suppressor genes, among them *p16*, *MLH1*, *VHL*, and *E-cadherin *[33]. Whether gene promoter hypermetylation is the cause or consequence of tumor suppressor gene silencing remains a matter of controversy; nevertheless, these views are not mutually exclusive. That DNA methylation is causal has been shown by the ability of diverse pharmacologic compounds and molecular techniques to reactivate gene expression upon inhibition of DNA methylation in cancer cells [34]. On the other hand, other findings suggest that hypermethylation-induced gene silencing could be associated with changes that determine gene expression, such as chromatin modification, so that methylation aids in maintaining the gene's silenced status. Strong support for the second view has derived from experiments showing that histone H3 lysine 9 methylation – that is, chromatin modification – occurred along with *p16 *re-silencing in absence of DNA methylation in cells in which *p16 *had previously been activated by DNA methyltransferase knock-out [35], and by data demonstrating *p16 *silencing in mammary epithelial cells that had escaped senescence and demethylated the promoter [36].

### Silencing of genes involved in tumor-host interaction

Although cancer cells are less immunogenic than pathogens, the immune system is clearly capable of recognizing and eliminating tumor cells. However, tumors frequently interfere with immune response development and function through several mechanisms such as loss of antigen processing and presentation, the Fas counterattacking system, escaping from death receptor signaling, engaging in inhibition-blocking activation, suppression of antitumor responses by regulatory T cells, and tumor-induced immune suppression [37]. Current research demonstrates that epigenetic defects are involved in at least some mechanisms that preclude mounting a successful host-antitumor response, including the HLA system, tumor-associated antigens, and accessory/co-stimulatory molecules.

Among tumor-associated antigens, testis-associated genes (CTA) are a sub-group of tumor antigens with restricted expression in testis and malignancies; CTA include the MAGE, NYESO, and SSX gene families and the GAGE/PAGE/XAGE superfamilies [38]. Distinct CTA can encode for different antigenic peptides that are presented to the immune system in association with various human leukocyte antigens (HLA), HLA class I or HLA class II allospecificities, eliciting both cytotoxic T lymphocytes (CTL) and humoral immune responses. De Smet et al., first showed a link between genomic DNA hypomethylation and *MAGE-1 *gene expression [39], and subsequent studies demonstrated direct correlation between demethylation of CpG at the promoter of several cancer testis antigen coding genes with their expression [40-43]. Surprisingly, 5-aza-2'-deoxycytidine demethylation treatment led to induction of *MAGE-, GAGE-, PAGE-*, and *XAGE-*type genes in tumor cells but not in normal cells [44].

Presentation of antigens within the context of HLA molecules is crucial both during T-cell priming and the effector phase of an adaptive immune response. Alterations in antigen processing and presentation are commonly observed in malignancies. In tumors, the antigen presentation pathway is commonly disrupted as a consequence of the mutations and/or deletions of one or several genes encoding components of the antigen-processing machinery, including proteasomal subunits and transporters peptides; thus, complete HLA loss is a common event in several murine and human tumors [45]. As for CTA, DNA methylation participates in regulation of the expression of the three classes of human leukocyte antigen class I antigens: HLA-A; HLA-B, and HLA-C, which are CpG-rich at their gene promoters. Nie et al. showed down-regulation of HLA class I antigens in esophageal carcinoma as a common mechanism for transcriptional inactivation caused primarily by DNA hypermethylation [46], as well as in melanoma, where 5-aza-2'-deoxycytidine significantly enhances the constitutive expression of HLA class I antigens, of HLA-A1 and -A2 alleles, and of the co-stimulatory molecule, intercellular adhesion molecule-1, and lymphocyte function-associated antigen-3 [47]. Further, it was shown that 5-aza-2'-deoxycytidine leads to re-expression of HLA class I antigens and restoration of antigen-specific CTL response in melanoma cells [48]; likewise, it has been shown that methylation-induced silencing of the class II transactivator (CIITA) leads to lack of HLA-DR induction in hematopoietic tumor cells and solid tumors such as gastric and colorectal carcinomas [49,50].

### Current epigenetic therapy agents

Reactivation of tumor suppressor genes silenced by an epigenetic mechanism such as gene promoter methylation is a very attractive molecular target for cancer therapy. There are several demethylating agents currently being evaluated in pre-clinical and clinical studies (Table 1). The classical demethylating agents comprise the analogs of deoxycytidine, including 5-azacytidine, 5-aza-2-deoxycytidine, 1-β-D-arabinofuranosil-5-azacytosine, and dihydro-5-azacytidine. 5-azacytidine and its analog 5-aza-2-deoxycytidine are the most frequently studied and were developed over 30 years ago as classical cytotoxic agents, but were subsequently discovered to be effective DNA methylation inhibitors [51]. These were tested as such in several phase II studies against solid tumors, demonstrating very modest activity [52]. Contrariwise, their antileukemic activity was very promising, and both are being revived due to their demonstrated inhibitory activity upon DNA methylation and gene-reactivating function.

**Table 1:** Agents targeting DNA methylation and histone deacetylase in preclinical and clinical development

**DNA methylation inhibitors**
*deoxycytidine analogs:*
5-azacytidine, 5-aza-2-deoxycytidine, 1-β-D-arabinofuranosil 5-azacytosine, dihydro- 5-azacytidine
*nucleic acid-based:*
MG98 antisense oligonucleotide
*cytidine deaminase analogs:*
zebularine
*non-nucleoside analogs:*
(-)-epigallocatechin-3-gallate, procaine, procainamide, hydralazine, RG108, psammaplins

**Histone deacetylase inhibitors**
*Small molecular weight carboxilates:*
sodium butyrate, valproic acid, sodium phenylbutyrate and pivaloyloxymethyl butyrate
*Hydroxamic acids:*
SAHA, trichostatin A, SBHA
*Benzamides:*
CI-994, MS-275
*Epoxyketones:*
trapoxin B, 2-amino-8-oxo-9,10-epoxydecanoic acid
*Cyclic peptides:*
apicidin, depsipeptide
*Hybrid molecules:*
CHAP 31, CHAP50
*Miscellaneous:*
Cyclostellettamines, Carbamazepine

Adapted from reference 106.

Currently, 5-azacytidine is Federal Drug Administration (FDA)-approved for use against myelodysplastic syndrome, and 5-aza-2-deoxycytidine is also being tested in myelodysplastic syndrome and in a variety of solid tumors due to its DNA demethylating properties [53]. In particular, 5-aza-2-deoxycytidine when infused for 10 days proved to be well-tolerated and induced responses in 65% of 50 patients with acute myelogenous leukemia, myelodysplasia, chronic myelogenous leukemia, and acute lymphocytic leukemia [54]. Despite poor activity against solid tumors of these nucleoside analogs, it is remarkable that proof of the concept concerning the ability of DNA methylation compound inhibitors to demethylate and reactivate tumor suppresor gene expression has been shown in solid tumors [55]. Whether or not the reactivating effect can translate into a clinical response on its own or in combination with classical cytotoxic therapies remains to be demonstrated.

As a second category of demethylating agents, there is the antisense oligonucleotide MG98 against the 3' untranslated region of *DNMT1 *mRNA that codes for the enzyme DNA methyltransferase 1, which is responsible for DNA methylation maintenance [56]. This agent has shown an ability to inhibit *DNMT1 *gene expression without affecting other *DNMTs *and to cause demethylation with *p16 *re-expression in bladder and colon cancer cell lines, as well as to produce tumor growth inhibition in nude mice bearing human lung and colon xenografts [57]. MG98 has been evaluated in a phase I trial in patients with advanced or refractory solid tumors and has demonstrated its tolerability, despite the fact that dose-limiting toxicities of transaminitis, thrombocytopenia, and fatigue prohibited higher doses. MG98's molecular efficacy was demonstrated by producing a *DNMT *mRNA level decrease in the peripheral blood cells in six of 10 patients [58].

The fact that deoxycytidine analogs, like current cytotoxic agents, are not only carcinogenic but also exhibit neutropenia as their dose-limiting toxicity even when used at doses required for demethylation [59] has renewed interest in finding effective and less toxic demethylating agents. Zebularine is a new oral cytidine analog originally synthesized as a cytidine deaminase inhibitor that has shown to cause demethylation and reactivation of a silenced and hypermethylated *p16 *gene in human bladder tumor cells grown in nude mice. Zebularine was also shown to be minimally cytotoxic *in vitro *and *in vivo *and can be administered continuously at a lower dose to maintain demethylation for a prolonged period, possible only because of its low-toxicity profile; to date, there are no results of clinical trials with this agent [60]. Within this class of so-called "non-toxic and orally administered agents", there is the green tea major polyphenol (-)-epigallocatechin-3-gallate (EGCG) that demonstrated to be an effective *DNMT *activity inhibitor at micromolar concentrations and that was able to demethylate and reactivate expression of several tumor suppressor genes such as *p16*, *RAR-β2*, and *MGMT *in cancer cell lines [61].

There is another class of so-called "old drugs" that includes hydralazine. The demethylating activity upon gene promoters of tumor suppressor genes of these compounds was recently demonstrated. Procainamide, a non-nucleoside inhibitor of DNA methyltransferases approved for treatment of cardiac arrhythmias, can demethylate the *GSTP1 *promoter, a common somatic genome change in human prostate cancer, and reactivates expression of the gene *in vitro *and in nude mice [62]. A related drug, procaine, possesses the ability to demethylate and reactivate tumor suppressor gene expression, such as the *RARβ2 *gene, in a breast cancer cell line, an effect accompanied by growth-inhibitory actions [63].

### Hydralazine as a demethylating agent

Clues to underscoring hydralazine's DNA demethylating property have derived from its ability to induce a lupus-like syndrome in connection with studies demonstrating DNA methylation disorder participation in systemic lupus erythrematosus (SLE).

### Lupus-like syndrome induced by hydralazine

More than 80 drugs have been associated with drug-induced lupus erythrematosus [64]; at least 39 of these are currently in use [65]. Only two of these, procainamide and hydralazine, are considered as high risk for induction of the syndrome, with up to 8 and 20% frequency respectively, during 1 year of therapy at currently employed doses [64,65]. Hydralazine was introduced as an antihypertensive drug in 1952, and the first case of hydralazine-induced lupus was reported soon thereafter [66]. Prevalence of slow acetylators among patient populations at risk for developing lupus erythrematosus is obviously much greater than the disease prevalence. Only 1–3% of populations of which at least 50% are slow acetylators develop hydralazine-induced lupus. In a study of 26 patients with hydralazine-induced lupus, 25 were slow acetylators, with a 4:1 ratio of females to males; this discrepancy may be explained by the fact that additional genetic traits are important in predisposing individuals to lupus [67,68].

Clinical features and laboratory manifestations of drug-induced lupus are similar to those of lupus erythrematosus. Symptom onset can be slow or acute, although an interval of 1–2 months typically occurs before diagnosis is made. As with idiopathic lupus, the most commonly observed abnormality is the presence of anti-nuclear antibodies, and these autoantibodies usually react with chromatin; however, unlike in patients with lupus, anti-chromatin antibodies rarely react with native (double-stranded DNA. In summary, neither the clinical nor the serologic picture are specific for drug-induced or idiopatic lupus; hence, the key (albeit retrospective) diagnostic tool is the observation that drug-induced lupus symptoms usually resolve within days to weeks after discontinuing the offending drug without introduction of anti-inflammatory medications [64].

### Mechanisms of hydralazine-induced lupus syndrome

Early studies suggested that interaction between hydralazine with thymidine and deoxycytidine was at least partly responsible for initiating the syndrome [69], and that an immune response to hydralazine may be important in human hydralazine-induced SLE [70].

Additional recent studies have strongly supported that SLE pathogenesis is strongly related with disorders in DNA methylation, beginning with an observation by Richardson et al. that CD8 T cells treated with 5-aza-cytidine – but not with hydroxyurea, a DNA synthesis inhibitor – re-expressed CD4, implying that DNA methylation may be one of the mechanisms involved in thymocyte maturation [71]. Subsequently, the authors showed that the 5-azacytidine-treated cloned, CD4 IL-2- dependent antigen-specific T cells lost the requirement for antigen and could be activated by autologous macrophages [72]. Autoreactivity correlated with over-expression at a transcriptional level of lymphocyte function-associated antigen-1 (LFA-1) [73], and T cells overexpressing this molecule become auto-reactive and caused a lupus-like disease in syngeneic mice [74] as it did in mice injected with syngeneic normal murine T cells treated with demethylating drugs [75,76]. Latter, it was shown that 5-azacytidine, procainamide, and hydralazine induce CD11a promoter demethylation and re-expression [77]. Further, it has been demonstrated that patients with rheumatoid arthritis and SLE had DNA hypomethylation of T cells [78], which correlates with decreased DNA methyltransferase enzyme activity and DNMT1 mRNA due to inhibited ERK pathway signaling [79]. In addition, lymphocytes from patients with lupus had at least over-expression and promoter demethylation of genes coding for CD11a [80] and CD70, a B cell co-stimulatory molecule [81]. In summary, these studies indicate that DNA hypomethylation and deregulated expression of molecules including LFA-1 and CD70 are fundamental for the pathogenesis of both drug-induced and idiopathic human lupus.

### Hydralazine in demethylating and reactivating tumor suppressor genes

**Figure 1:**
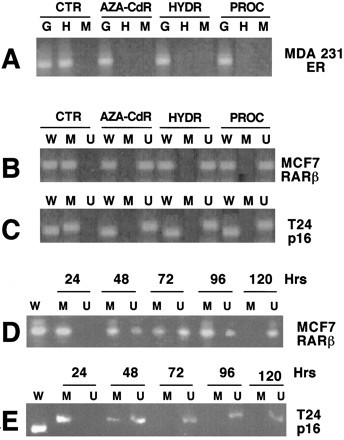
Methylation analysis of the ER, RARβ, and p16 genes. **A**, G is undigested genomic DNA as positive control, H is HpaII, and M is MspI. The band in H of control lanes indicates lack of digestion with HpaII and, therefore, methylation of untreated MDA-231 cells. **B **and **C **show the analysis by methylation-specific PCR of RARβ and p16 genes in MCF-7 and T24 cells. W is wild type, M is methylated primers, and U is unmethylated primers. D and E show a time course experiment of RARβ and p16 gene demethylation over 5 days on the MCF-7 and T24 cells, respectively. (Reproduced with permission from the AACR. Ref. 83. *Clin Cancer Res *2003, **9**:1596–1603).

**Figure 2:**
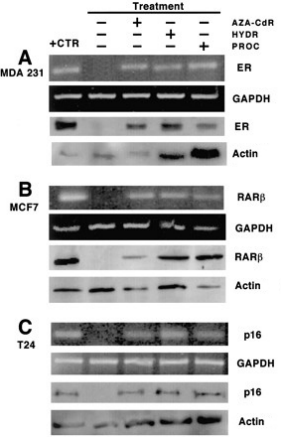
Expression of the mRNA and proteins of **A**, ER (MDA-231); **B**, RARβ (MCF-7); and **C**, p16 (T24) genes by RT-PCR and Western blot. The positive control (+ctr) for A is MCF-7, for B is MDA-231 exposed to 5-aza-CdR/atRA, and for C is HeLa cells. (Reproduced with permission from the AACR. Ref. 83. *Clin Cancer Res *2003, **9**:1596–1603).

**Figure 3:**
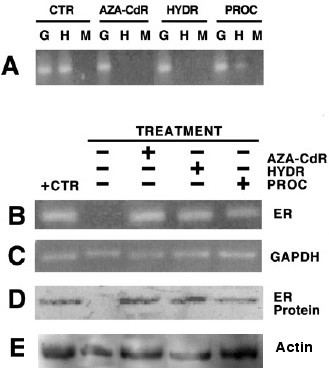
Analysis of methylation and product expression of the ER gene in nude mice. **A **shows that the tumor of control mice (CTR lanes) was methylated as shown by the band in the Lane H indicating no digestion with HpaII. The band disappeared with the treatment with 5-aza-CdR and hydralazine, which indicates gene demethylation. In PROC (procainamide) lanes, the band is absent in MspI but is very weak in HpaII, indicating that was only partially demethylated; **B **and **D **are the product expression by RT-PCR and Western blot. In both cases, the intensity of bands in procainamide is weaker, correlating with a partial demethylation, (**C **and **E **are the loading controls, GAPDH and actine respectively). G is undigested genomic DNA. (Reproduced with permission from the AACR. Ref. 83. *Clin Cancer Res *2003, **9**:1596–1603).

These observations, unrelated to the field of DNA methylation in cancer and demonstrating that hydralazine possesses DNA demethylating activity [82], led to testing the hypothesis that hydralazine, by virtue of its DNA methylation inhibitory activity, could restore tumor suppressor gene re-expression by relieving hypermethylation at the gene promoter. To that end, demethylation and re-expression of tumor suppressor genes, estrogen receptor, and *p16, RARβ*, which are methylated in MDA-231, T24, and MCF-7 cell lines, respectively, were studied. Treatment of these cell lines with hydralazine at 10 μM for 5 days led to gene promoter demethylation at these genes, which correlated with gene re-expression at the level of RNA and protein levels (Figures 1 and 2); in addition, it was shown that the reactivated gene product was functional. Hydralazine's gene-demethylating and -reactivating effects were also observed for the estrogen receptor gene in estrogen receptor-negative MDA231 cell-xenographed mice treated for 7 days with hydralazine by intraperitoneal (i.p.) route at doses equivalent to those observed in hypertensive patients [83] (Figure 3).

**Figure 4:**
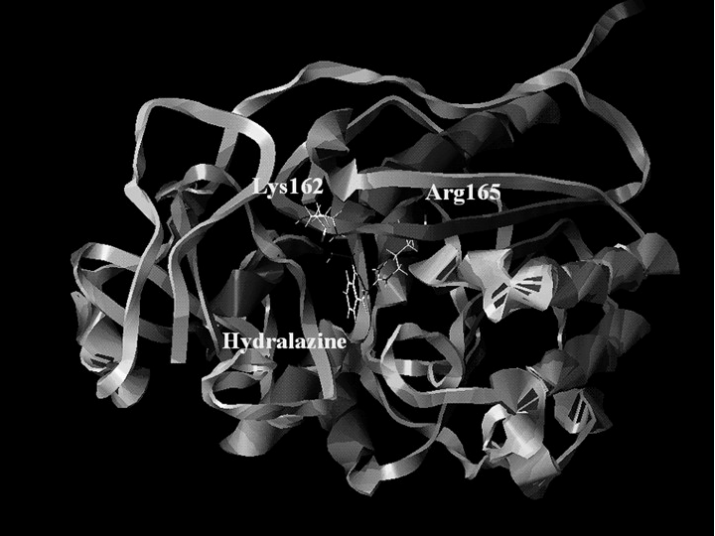
Docking between the enzyme DNA methyltransferase and hydralazine showing the residues at the pocket (Lys 162 and Arg 165) that interact with the hydralazine molecule (Modified from ref. 84. *Lett Drug Design Discov *2005, **2**:282–286).

**Figure 5:**
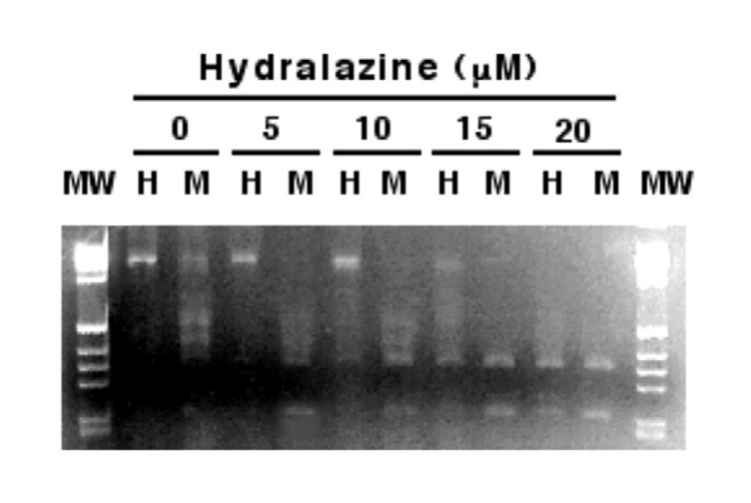
In *in vitro *methyltransferase assay of hydralazine. There was no digestion with HpaII (lanes H, with 0 and 5 μM of hydralazine) indicating full methylation. Starting at 10 μM of hydralazine, bands in the lanes digested with HpaII (H) are visible, and at 20 μM the pattern of bands in HpaII (H) and MspI (M) digestions is similar indicating full demethylation. (MW: molecular weight). The substrate DNA for the *in vitro *methylation assay was a 1112 bp fragment of the type I Human Herpes Simplex virus tymidine kinase gene which has a high GC content. The methylation reaction contained 1 μg of substrate DNA and 10 units of M.SssI methylase (0.5 μmol/L, New England Biolabs, Beverly, MA) in a final volume of 30 μL. Hydralazine was added to final concentrations of 0, 5, 10, 15, and 20 μmol/L starting two hours before adding the M.SssI enzyme for the methylation reaction. Reactions were done at 37°C for 2 hours. After completion, the reaction the DNA was purified using the rapid PCR Purification system (Ijamsville, MD). Then, equal volumes of extracted DNA for each sample were placed in two separated eppendorf tubes for restriction enzyme digestion (one tube with HpaII and the other with MspI) both from New England Biolabs. Each reaction contained 10 U of the enzyme, in a final reaction volume of 50 μL at 37° for 3 hours. After digestion, samples were reduced to dryness by speedvac concentration and redissolved in 10 μL of ddH20 and then loaded into a 2% Tris-borate EDTA agarose gels for electrophoresis.

**Figure 6:**
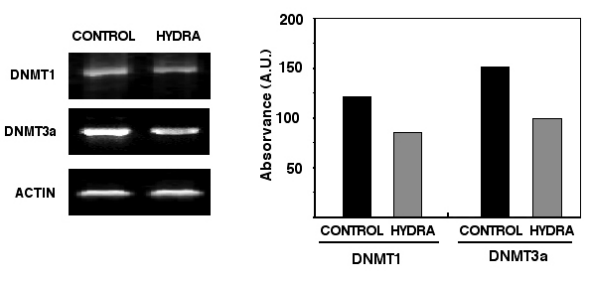
Expression of DNMT1 and DNMT3a in MCF-7 cells. The expression of DNMT1 and DNMT3a mRNA is decreased in MCF-7 cells treated with hydralazine. The lower band intensity was confirmed by densitometric (right) analysis, (control is untreated cells). MCF-7 cells were growth arrested for 48 hours by serum deprivation, then treated for 24 hours with hydralazine at 10 μM and then RNA extracted for analysis. Reverse transcription was carried out as previously described [110] using random hexamers, superscript II reverse transcriptase (Life Technologies) and 2.5 μg of total RNA as recommended by the manufacturer in a total volume of 50 μL. One microliter of RT reaction was used for subsequent PCR amplification for each of the desired transcripts with dNTPs and Amplitaq (Applied Biosystems). Primer sequences used were as follows: DNMT1 sense 5-gat cga att cat gcc ggc gcg tac cgc ccc ag-3 and antisense 5-atg gtg gtt tgc ctg gtg c-3. DNMT3a sense 5-ggg gac gtc cgc agc gta cac-3 and antisense 5-cag ggt tgg act cga gaa atc g-3. Amplification conditions were: 94 C° for 5 min 1 cycle; 94 C° 30 sec, (58°C for DNMT1 and 65°C for DNMT3a) annealing temperature for 1 min, and 72 C° for 30 sec, 35 cycles.

With the aim of explaining hydralazine's demethylating effects, we assessed the structural and electronic properties between hydralazine and methyltransferase by modeling the most stable conformation of hydralazine and the pocket at the active site of the enzyme. Results indicate the existence of a high-affinity interaction between hydralazine and the DNA methyltransferase. The residues Lys 162 and Arg 240 within the enzyme active site demonstrated four interacting points with hydralazine (Figure 4) at distances between these residues and hydralazine nitrogen atoms not exceeding 4A°. These interactions are energetically stable, supporting that hydralazine may inhibit DNA methyltransferase [84]. These results from modeling have been confirmed by experiments demonstrating the ability of hydralazine at concentrations between 10 and 20 μM to inhibit DNA methylation in an *in vitro *DNA methyltransferase assay (Figure 5). It remains debatable whether or not hydralazine directly inhibits DNA methyltransferase enzymatic activity. In fact, Richardson's group failed to demonstrate enzymatic inhibition *in vitro *with hydralazine; instead, the authors reported that hydralazine decreased RNA methyltransferase 1 and 3a expression in a similar manner to PD98059, a mitogen-activated protein kinase kinase (MEK) inhibitor, this suggesting that hydralazine does not directly inhibit DNA methyltransferase enzymatic activity [85]. These discrepancies with regard to hydralazine's precise mechanism of action as DNA methylation inhibitor may stem from the different DNA methyltransferase assays used. Richardson's group employed a methyltransferase assay with a partially purified enzyme obtained from Jurkat T cell lysates assayed upon hemimethylated double-stranded oligonucleotides and ^3^H-AdoMet [85], whereas our group utilized an assay based on lack of DNA restriction after the DNA substrate was treated with M. SssI methylase enzyme; this latter assay has been used recently to characterize the demethylation inhibitory activity of newer demethylating compounds [86]. Nevertheless, the issue concerning this direct inhibition by hydralazine on the enzyme needs to be further addressed. It is remarkable, however, that our group likewise Richardson's group, also have observed decreased expression of DNMT1 and DNMT3a, but no DNMT3b mRNA by RT-PCR in MCF-7 cells after treatment with hydralazine (Figure 6).

A recent study from the Jones group compared the DNA-methylating and gene-reactivating activity of 5-aza-2'-deoxycytidine against non-nucleoside inhibitors (-)-epigallocatechin-3-gallate, hydralazine, and procainamide. These authors found that 5-aza-2-deoxycytidine is far more effective both in its DNA methylation inhibitory activity and in its ability to reactivate methylation-silenced genes in cancer cells [87]; nonetheless, the authors failed to reproduce the findings obtained by the three independent researcher groups who reported the effect of these non-nucleosides upon DNA methylation. Contrariwise, in a recent report the ability of hydralazine to demethylate and reactivate estrogen receptor gene expression was confirmed [88].

### Antitumor efficacy in pre-clinical hydralazine and valproic acid models

Due to the well-known synergistic effect of DNA methylation and histone deacetylase inhibitors on gene re-expression and antitumor effects, the combined effect of hydralazine and valproic acid has been investigated in pre-clinical models. Results demonstrate that treatment with these drugs in combination possesses a strong growth inhibitory effect in several cell lines tested. Nevertheless, hydralazine alone at the concentration tested had no or small growth-inhibitory properties. With the exception of 5-aza-2'-deoxycytidine, a classical cytotoxic agent with demethylating properties [53], the *in vivo *or *in vitro *antiproliferative effects of other DNA demethylating agents such as procainamide, zebularine, tea polyphenol (-)-epigallocatechin-3-gallate, and RG108 [86] are low or absent at DNA demethylating concentrations. Interestingly, MCF-7 and SW480 were more sensitive to the combination of compounds, although the difference was statistically significant only in SW480 cells [89] suggesting that the known synergistic effect on tumor suppressor gene re-activation by a DNA demethylating agent and a HDAC inhibitor [90] lead in some models to stronger antitumor effects [91,92].

Data on the synergy between a DNA methylation inhibitor and an HDAC inhibitor for gene re-expression led to exploration of the effect of hydralazine plus valproic acid on global gene expression. Microarray analysis utilizing the Amersham CodeLink system containing 55K genes probes demonstrated that 1,281 genes (651 up-regulated and 630 down-regulated) were differentially expressed at least three-fold differently from an untreated control. Among up-regulated genes, 153 were specifically induced by hydralazine and 178, by valproic acid; however, when cells were exposed for 4 days to hydralazine and then for 16 h to valproic acid the number increased to 352, demonstrating that these epigenomic active drugs have a synergistic effect upon gene expression [89]. Synergy between DNA methylation inhibitor 5-aza-2'-deoxycytidine and deacetylase inhibitor trichostatin A on gene re-expression at a global scale is already known; it has been shown that the percentage of up-regulated genes with 5-aza-2'-deoxycytidine was 1.9, 1.1 with trichostatin A, but 10.4% with the combination [93], indicating that reversal of two epigenetic factors (i.e., DNA demethylation and histone hyperacetylation) synergizes gene re-expression [94]. Among genes up-regulated by hydralazine and valproic acid are those coding for major histocompatibility complex, class I-related and major histocompatibility complex, class II, DR alpha. This class I-related antigen is a powerful NKG2D ligand for NK cell-mediated antitumor immunity induction, whereas the DR alpha chain is crucial for antigen presentation within the context of class II MHC [95,49,50].

**Figure 7:**
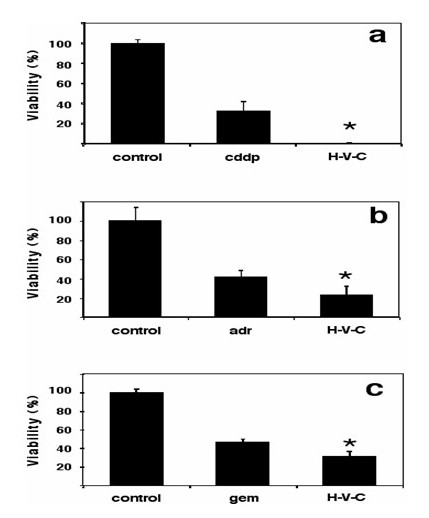
HeLa cells were treated for 24 hours with panel **a**: cisplatin (cddp), panel **b**: adriamycin (adr) or panel **c**: gemcitabine (gem) at or close to IC^50 ^plus hydralazine (h) and valproic acid (v). Afterwards medium was removed and fresh medium containing only hydralazine and valproic acid was added for additional 48 hours after which viability was measured. There was a significant higher cytotoxicity of the chemotherapeutic agent when used in combination with hydralazine plus valproic acid. Cisplatin alone at 12 μM caused a reduction in viability to 37% in HeLa cells which was essentially zero viability when cells were co-treated with hydralazine plus valproic acid (h-v-c). The increasing toxicity of the combination was also seen with adriamycin and gemcitabine. Viability was 42% and 45% in the chemotherapy drugs alone versus 27% and 37% respectively when hydralazine plus valproic acid were added to chemotherapy drugs (h-v-c). (Reproduced from ref. 89. *Cancer Cell Int *2006,**6**:2).

Upon any stimuli, cells must orchestrate a number of changes in gene expression, as well as in other dynamic processes such as protein phosphorylation, protein trafficking, and protein-protein interactions, to cope with cytotoxic stress [96]. Therefore, it is vital for cells to possess intact transcription regulation machinery. On this basis, it was hypothesized that malignant cells exposed to cytotoxic chemotherapeutic drugs would have a deranged response and that this would perhaps compromise survival if co-treated with drugs targeting two of the main gene-regulating processes: DNA methylation and histone acetylation. In fact, hydralazine and valproic acid increased the cytotoxicity of chemotherapy agents cisplatin, doxorubicin, and gemcitabine (Figure 7) [89]; it is noteworthy, however, that sensitization was higher for cisplatin and doxorubicin, as could be expected because loosening the chromatin structure by histone acetylation increases the efficiency of anticancer drugs that target DNA [97]. The observed chemosensitization *in vitro *was evaluated in a murine model of fibrosarcoma using nude mice xenografted with the sarcoma cell line HT1080. Remarkably, while tumors receiving only adriamycin re-grew 1 week after the adriamycin final dose, animals treated with hydralazine plus valproic acid showed no tumor re-growth [89]. These results suggest that the combination's antitumor efficacy was higher and that therefore no microscopic residual remained or alternatively, that the treatment disabled cell proliferative capacity. This in principle may be particularly relevant for patients with minimal residual disease after curative attempt by surgery, chemotherapy, and/or radiotherapy.

### Clinical studies of hydralazine and valproic acid in patients with cancer

**Figure 8:**
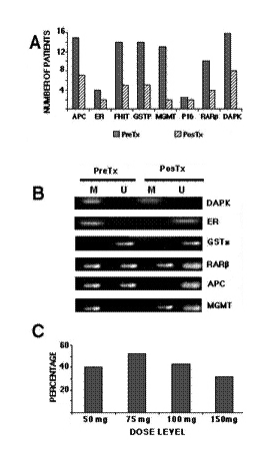
**A**. Pre- (dark bars) and post-hydralazine treatment (light bars). The bars represent the number of patients that showed methylation for each studied gene from each of the 16 patients. **B**. Representative cases of genes (M methylated, U unmethylated; pre/post): M/M; M/U; U/U, M/U; MU/U; M/ U-M. **C**. Percentage of demethylation after treatment according to the dose. Percentage was calculated considering 100% methylation the total number of pre-treatment methylated genes in each cohort of 4 patients (Reproduced from ref. 74. *BMC Cancer *2005, **5**:44).

These pre-clinical studies were the foundation for performing a phase I study to investigate whether hydralazine administered by oral route at doses commonly used for treatment of high blood pressure could demethylate and re-activate tumor suppressor gene expression in the tumors of patients with cervical cancer [98]. The study was performed in 16 newly diagnosed patients with cervical cancer with locally advanced disease receiving 50, 75, 100, or 150 mg a day for 10 days. The treatment was well-tolerated and methylation analysis of the biopsies taken prior to and after 10 days of hydralazine administration was performed in all patients. Overall, 70% (89 of 128) of pre-treatment samples analyzed (eight genes for each of the 16 pre-treatment biopsies) had at least one methylated gene, and all 16 patients had at least one methylated gene. Irrespective of the hydralazine dose administered, post-treatment biopsies showed a gene-variable demethylation rate varying from 15% (2/13 samples) for the *MGMT *gene to 67% of demethylation for the *p16 *gene. Correlation between demethylation analysis and dose level revealed the following: at 50 mg a day, 40% of methylated genes suffered demethylation; at 75 mg, this was 52%; at 100 mg, the rate was 43%, and the rate was 32% for the 150-mg dose (Figure 8).

**Figure 9:**
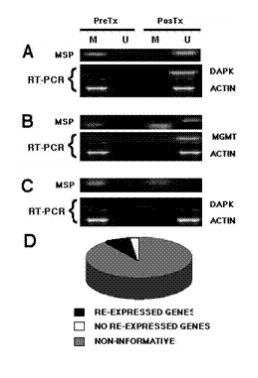
Representative cases correlating methylation and re-expression before and after hydralazine treatment. **A **is a patient treated with 75 mg/day that demethylated and re-expressed the DAPK gene. **B **corresponds to a patient receiving 150 mg/day who showed only the methylated band pre-treatment, but both bands after treatment, which correlated with re-expression of MGMT. **C **is a 50 mg/day patient which failed to demethylate the DAPK gene and therefore lacked expression. D represents the distribution of informative cases. From the 128 genes/cases, 116 were RT-PCR positive regardless of the methylation status, hence were not informative. In the remaining 12 cases, nine demethylated and re-expressed the gene. (Reproduced from ref. 74. *BMC Cancer *2005, **5**:44).

Regarding expression of the analyzed genes, 90% (116 of 128) of tumor samples expressed the messenger in the pre- and post-treatment biopsies regardless of methylation status; hence, these were not informative. Notwithstanding this, nine of 12 informative cases had the gene re-expressed after treatment: one case at 50 mg; four cases at 75 mg; one case at 100 mg, and three cases at 150 mg (Figure 9). Interestingly, at the doses tested there were no observed changes in the methylation status of imprinted and "normally methylated" genes in the DNA of peripheral blood cells. Additionally, hydralazine lowered global methylcytosine content by 1% in tumors, as evaluated by a capillary electrophoresis method [99].

It is widely accepted that cancer development has been associated with DNA methylation deregulation and aberrant histone deacetylase (HDAC) activity, which cooperate for inducing aberrant gene silencing [100]. Therefore, a number of investigation groups are currently exploring the clinical antitumor activity of a DNA methylation inhibitor plus a histone deacetylase inhibitor [101].

Among histone deacetylase inhibitors, the discovery that valproic acid, which belongs to the short-chain fatty acids category and which showed to be an effective HDAC inhibitor [102,103], encouraged investigation of this agent as a potential cancer therapy agent. We recently demonstrated, within the context of a phase I study, that magnesium valproate when used at only slightly higher doses than those utilized as anticonvulsant, not only produces H3 and H4 histone hyperacetylation in PBMN cells, but also leads to hyperacetylation of H3 and H4 hyperacetylation and HDAC activity inhibition in primary tumors of patients with cervical cancer [104]. These data have led us to investigate the clinical efficacy of hydralazine and magnesium valproate in a series of on-going phase II studies.

Breast cancer exhibits a number of epigenetic alterations that potentially can be reverted by epigenetic therapies [105]; hence, in the first study newly diagnosed patients with locally advanced breast carcinoma receive four courses of neoadjuvant chemotherapy with adriamycin and cyclophosphamide plus hydralazine and magnesium valproate on a daily basis beginning 7 days before the first chemotherapy cycle and continuing through the end of chemotherapy, with pathologic response rate as the end point. Likewise, epigenetic alterations in cervical cancer are common [106]; hence we are performing a phase II trial in previously untreated FIGO stage IIIB cervical cancer. Patients receive standard weekly cisplatin plus radiation along with the combination of hydralazine and magnesium valproate with the aim of increasing clinical complete response rate.

On the other hand, studies have shown that DNA methylation and histone deacetylase inhibitors are able to resensitize drug-resistant tumors to established treatments [107]; hence, we are testing whether the combination of these epigenetic agents added to chemotherapy induces responses in common solid tumors such as breast, lung, cervical, ovarian, head & neck, prostate, and testicular carcinomas. In this trial, patients with measurable disease who exhibit non-response or tumor progression after three courses of palliative chemotherapy initiate with hydralazine and magnesium valproate plus the same chemotherapy scheme. Finally, we are also testing the clinical activity of the combination alone in myelodysplasic syndrome and refractory acute leukemia, because these tumor types are responsive to a combination of DNA demethylating and histone deacetylase inhibitors [108,109]. In all these trials, several parameters, including global and gene specific DNA methylation and histone acetylation, are being evaluated to gain insight into the predictive tumor response value of these molecular markers.

## Conclusion

Targeting DNA methylation and histone deacetylase constitutes a promising antitumor treatment, which is also denominated epigenetic therapy. The fact that the most extensively studied nucleosides with known DNA methylation inhibitory activity, 5-azacytidine and 5-aza-2'-deoxycytidine, exhibit myelosuppression even when used at low doses has spurred the search for less toxic alternatives. Among non-nucleoside demethylating agents, hydralazine is a good candidate for clinical development mainly due to the fact that its extensive use for decades for cardiovascular conditions has demonstrated its tolerability and safety. Existing data show that this agent is able to demethylate and re-activate tumor suppressor gene expression in patients with cancer. Its good tolerability also permits its use in combination with an epilepsy treatment-approved histone deacetylase inhibitor. The antitumor efficacy and safety of this "epigenetic therapy" are currently being evaluated in phase II studies, along with chemotherapy and/or radiation for a variety of malignancies in different scenarios. If encouraging, results of these trials will dictate its further testing in randomized phase III trials.

## Competing interests

The author(s) declare that they have no competing interests.

## Authors' contributions

CA and MC participated in writing sections of the review, B S-P, and E P-C conducted the laboratory work, and A D-G conceived of the review and wrote the manuscript.
